# Gait Phase Detection for Lower-Limb Exoskeletons using Foot Motion Data from a Single Inertial Measurement Unit in Hemiparetic Individuals

**DOI:** 10.3390/s19132988

**Published:** 2019-07-06

**Authors:** Miguel D. Sánchez Manchola, María J. Pinto Bernal, Marcela Munera, Carlos A. Cifuentes

**Affiliations:** Department of Biomedical Engineering, Colombian School of Engineering Julio Garavito, Bogota 111166, Colombia

**Keywords:** gait phase detection, inertial motion data, inertial measurement unit, force sensitive resistors, threshold-based algorithm, hidden Markov model, subject-specific training, standardized parameters training

## Abstract

Due to the recent rise in the use of lower-limb exoskeletons as an alternative for gait rehabilitation, gait phase detection has become an increasingly important feature in the control of these devices. In addition, highly functional, low-cost recovery devices are needed in developing countries, since limited budgets are allocated specifically for biomedical advances. To achieve this goal, this paper presents two gait phase partitioning algorithms that use motion data from a single inertial measurement unit (IMU) placed on the foot instep. For these data, sagittal angular velocity and linear acceleration signals were extracted from nine healthy subjects and nine pathological subjects. Pressure patterns from force sensitive resistors (FSR) instrumented on a custom insole were used as reference values. The performance of a threshold-based (TB) algorithm and a hidden Markov model (HMM) based algorithm, trained by means of subject-specific and standardized parameters approaches, were compared during treadmill walking tasks in terms of timing errors and the goodness index. The findings indicate that HMM outperforms TB for this hardware configuration. In addition, the HMM-based classifier trained by an intra-subject approach showed excellent reliability for the evaluation of mean time, i.e., its intra-class correlation coefficient (ICC) was greater than 0.75. In conclusion, the HMM-based method proposed here can be implemented for gait phase recognition, such as to evaluate gait variability in patients and to control robotic orthoses for lower-limb rehabilitation.

## 1. Introduction

Human gait refers to the physiological way of locomotion which can be altered by several pathologies [1]. Gait analysis is of great help to therapists who wish to monitor the recovery of patients going through rehabilitation processes [2]. Within clinical settings, gait classification can be implemented as part of the control parameters for functional electrical stimulation (FES) [3,4], the detection of abnormal gait pattern in patients with paretic limbs and their classification based on known pathologies [5], and an estimation of the risk that elderly people fall [6]. Additionally, an atypical gait pattern can be an indicator of the progression of neurological disorders. For instance, atypical gait patterns have been proven to predict if seniors will develop dementia or cognitive decline [7]. Finally, researchers have managed to program humanoid robots to use human-based gait trajectories generated via gait classification [8], as well as consistently control wearable assistive devices such as robotic prostheses [9] and orthoses [10] for the recovery of lower-limb mobility.

In regards to gait partitioning, one may consider different granularities, i.e., number of involved gait phases. The main nomenclature divides the gait cycle into two events, namely, stance and swing phases. A two-phase model has proven to be sufficient to control the knee module of an active orthosis whose actuation is only performed at the beginning of these gait subsections [11]. Nonetheless, the most widespread approach relies on a four-phase model, which comprises: (i) the initial foot contact (IC) with the ground or Heel Strike (HS); (ii) the loading response phase or Flat Foot (FF); (iii) the heel lifting or Heel-Off (HO); and (iv) the initial Swing Phase (SP) or Toe-Off (TO) [12]. This gait granularity has been used for the actuation of multiple robotic ankle-foot orthoses (AFO) [13,14]. Since such devices are mainly implemented in patients with drop-foot, their control systems behave in the following manner: the actuation mechanism prevents foot slap in HS, minimizes the exerted impedance so that plantar flexion movements are not hindered during FF, and lifts the user’s foot to prevent toe drag in the case of SP [15].

Regardless of the detection method, various technologies are used to capture gait phases. On the one hand, non-wearable sensors, such as force platforms and motion-capture-based systems, set the benchmark in accuracy for walking kinematics [16]. However, such sensors are expensive and limited to indoor use [17]. For this reason, wearable sensors, such as ultrasonic sensors [18], electroneurogram (ENG) signals [4], footswitches [17,19,20,21], electromyography (EMG) signals [22] and inertial sensors [5,23,24,25,26], have become popular due to their affordability, shorter donning/doffing times, and less complex post-processing. Among wearable sensors meant for gait segmentation, foot pressure insoles or footswitches are considered the best detection option, since each gait phase can be related to a specific pressure pattern [19,20,21,25,27,28,29,30], and the footswitch-based reference has been widely adopted in the identification of gait events [8,11,22,31,32,33,34,35]. For flexible pressure sensors that are applicable to insoles, a polymer thick film called force-sensitive resistor (FSR) and piezoresistive sensors (e.g., FlexiForce), experience changing resistances as a function of pressure, are inexpensive, and have a convenient input composition [19]. Nevertheless, their use in everyday activities is not recommended as they need to be placed at optimal locations to accurately detect gait phases, thus requiring an experienced professional to determine their optimal placement [36]. Additionally, pressure insoles must be tailored for each subject’s foot, which incur higher research costs, and are continuously exposed to tear and friction, which results in a shorter lifetime [37]. Therefore, the use of either whole inertial measurement units (IMU) (consisting of gyroscopes, accelerometers, and magnetometers), or the combination of such inertial sensing components, has risen lately. In the present study, we make use of such sensors thanks to their cost-effectiveness [38], and the fact that inertial quantities present typical waveform features during a gait cycle [12].

Studies have been conducted positioning IMUs on the waist [26], thigh [39], shank [23,34,40,41], and foot instep [3,42]. We considered the shank location due to low inter-subject variability [23] and the fact that there is less soft tissue movement compared to the thigh [40]. However, we opted for fastening the IMU to the foot instep because of the better performance that scalar classifiers have shown with the sensor placed on this location, even compared to other vectorial classifiers that involve further inertial sensors placed at different lower-limb locations [31]. The sagittal angular velocity of the foot also represents a suitable parameter, since gyro output exhibits periodic and repeatable pattern along the gait cycle [12].

The study presented here features a gait phase detection system (GPDS) that utilizes inertial motion data from a single IMU to detect four gait phases by means of two detection algorithms. The comparison of performance of a threshold-based (TB) algorithm and a machine-learning approach based on a hidden Markov model (HMM), both compared to reference values coming from a pressure-sensing custom insole, serves as a way of unveiling which detection methodology best suits this type of system while minimizing the number of sensors used.

## 2. Related Works

Several approaches have previously been proposed for the automatic segmentation of the gait cycle. Most of these differ from the present work in that it presents the comparison among a variety of well-known segmentation approaches with respect to FSR-based reference values from a considerable number of study subjects with both typical and pathological gait patterns. By doing so, the performance of such detection algorithms is assessed in a target population which, to the best knowledge of the authors, has not yet been addressed with this particular experimental setup, namely hemiparetic adults. Additionally, since the most suitable partitioning scheme is meant to be implemented on a wearable rehabilitation device, the use of an inter-subject training procedure for the machine-learning method is also explored throughout this study in order to avoid the time-consuming subject-specific training approach, and thus reduce its setup time within clinical settings.

Computational methods for gait phase recognition fall into two main categories. The first category is comprised of algorithms, which divide the gait phases based on the threshold selection of either raw or processed data [3,8,19,20,23,28,29,34,35,37,40,41,42,43]. Secondly, some machine-learning approaches have emerged in recent years to substitute the aforementioned techniques that rely on hand-crafted feature extraction. These adaptive methods extract patterns on the basis of Support Vector Machines (SVM) [5], Linear Discriminant Analysis (LDA) [30], Gaussian Mixture Model (GMM) [4,24], HMM [11,31,32,33,44,45,46], Artificial Neural Network (ANN) [25,47,48], and hybrid algorithms [49]. Among artificial intelligence schemes, HMM has demonstrated superior performance [12].

Several authors have applied rule-based algorithms, the most conventional detection method, to accurately detect different granularities using different types of inertial sensors. For instance, Catalfamo et al. [40] used a zero-crossing detection approach for the gait segmentation into IC and TO at different inclination levels, by using a gyroscope placed on the shank. This algorithm was evaluated for ten healthy subjects and showed an overall detection success of 98%. Likewise, Gouwanda and Gopalai [37] applied a similar zero-crossing technique in shank angular velocity signals to detect the two main gait phases in real-time. Although all gait events were found to have a latency of less than 125 ms, abnormal gait patterns were simulated on healthy subjects by the use of ankle and knee braces instead of recruiting pathological participants. Furthermore, Rueterbories et al. [3] utilized a feature-based algorithm to identify gait events from differential acceleration signals of the foot. Their results show an early detection capability with respect to the reference system for ten hemiparetic volunteers. In addition, Chia Bejarano et al. [23] placed inertial and magnetic sensors laterally on the shank to calculate the angular velocity in the sagittal plane and the shank flexo-extension angle. They sensed three gait phases from these signals: IC, TO and Mid-Swing (MS). This algorithm exhibited an accuracy of 87% with a maximum time delay of 52 ms for ten stroke patients with severe impairment. Finally, Behboodi et al. [34] also extracted characteristics from shank angular velocity signals to detect seven gait events with a maximum time difference of around 75 ms.

In contrast, it has been proven that the single supervised training of a continuous HMM, followed by a classification based on foot gyroscope signals, is sufficient to achieve an accurate detection rate for phase detection via machine-learning [31]. Among the studies in which an HMM has been implemented under such experimental conditions, the research study undertaken by Mannini and Sabatini [44] presents some similarities to the present one because the performance of a TB algorithm is compared with that of an HMM. High sensitivity and specificity values, together with reduced timing errors (<20 ms), were found during both walking and jogging conditions. However, only a limited sample of six healthy participants was considered for data acquisition. The online implementation of this method [46] also showed promising results, with a detection latency of 100 ms. Similarly, Abaid et al. [32] featured an online HMM-based algorithm deployed in both typically developed children and children with hemiplegia. Although the values for specificity and sensitivity do not stand out as the aforementioned results do, their proposed algorithm does not require high training times. Similarly, a distributed classifier that identifies gait events based on a hierarchical weighted decision from scalar HMMs was applied by Taborri et al. [31] to angular velocities of ten healthy subjects. This study suggests that the computational load, relative to distributed and scalar classifiers, is about 100 times lower than that exhibited by vectorial classifiers. The same author also developed an inter-subject training approach [33] to eliminate subject-specific procedures and the related processing times. This technique posed no significant difference to the intra-subject training, which encourages the implementation of such standardized parameters training (SPT).

A granularity of four gait phases was chosen based upon the fact that this system is intended to be employed in the medium term in the AGoRA lower-limb exoskeleton [50], a robotic orthosis that primarily actuates on the knee and ankle joints along the sagittal plane. As mentioned in Section 1, the amount of detected phases is more than sufficient to correct the drop-foot gait pattern at the ankle joint. This wearable robotic device is currently controlled by means of a TB algorithm (to be described in later sections), which is integrated into its control system in real-time, while mounted to the Robot Operative System (ROS), a meta-operative system widely used in robotic applications [51].

Taking into account the premises found in literature, this study proposes the validation of two algorithms (one of them trained by two different training approaches) for a GPDS that only uses the uni-axial gyro and accelerometer signals drawn from an embedded IMU placed on the foot instep to accurately detect four gait events. By using a reduced number of sensors, the proposed system poses as a smaller, lighter, and less expensive alternative with low power consumption for robotic orthotic devices.

## 3. Materials and Methods

### 3.1. Theoretical Approach

Two classification strategies have been implemented in the present work for the automatic identification of four gait phases, drawn from inertial data coming from a single IMU located at the foot instep. These partitioning strategies are popular in the technical field related to gait analysis [12]. The first and most easily implemented strategy is a TB algorithm that determines the gait phases of interest by establishing certain decision rules and thresholds (to be described in depth shortly), which must be met to jump from one gait phase to another. The above mentioned is the reason this method can also be considered a finite-state machine (FSM). The other partitioning method may be viewed as a machine-learning algorithm since it requires a training stage and a posterior testing stage [11]. Specifically, the implemented algorithm is based on a continuous HMM.

#### 3.1.1. Classification Using a Threshold-Based Detection Algorithm

The implemented threshold-based (TB) detection algorithm is similar to those developed by Rueterbories et al. [3] and Catalfamo et al. [40], whose studies use inertial sensors positioned on the volunteer’s shank to gather linear acceleration and angular velocity signals. Because the location of the sensors significantly affects the data collected, the rules that govern our detection algorithm are established based on the unique waveform that foot motion data present. In particular, only the normal component of the accelerometer signals ($A_{y}$) and the mediolateral axis rotation component in the case of the gyroscope ($G_{y}$) are fed to the TB algorithm as inputs, because the movement of lower-limb joints during walking occurs mostly along the sagittal plane. Time stamps are also used as algorithm inputs since this detection algorithm makes use of spatial thresholds as well as temporal limits.

**Figure 1:**
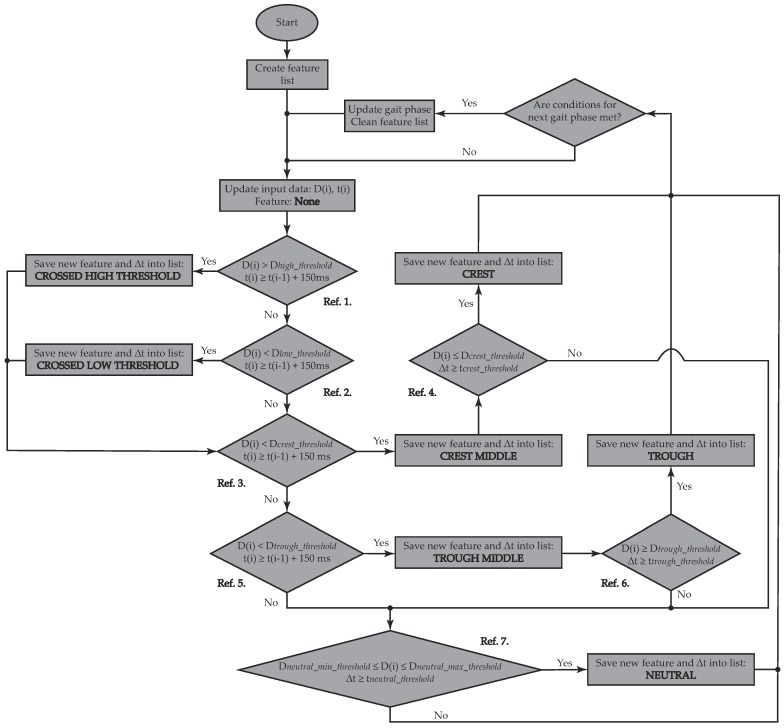
Flowchart of feature extraction from inertial motion data. This chart shows how a feature list is updated based on the fulfilment of certain conditions. The occurrence of each feature and their corresponding conditions are sequentially assessed in the following manner: Crossed High Threshold (Ref. 1), Crossed Low Threshold (Ref. 2), Crest Middle (Ref. 3), Crest (Ref. 4), Trough Middle (Ref. 5), Trough (Ref. 6), and Neutral (Ref. 7). When a new gait phase is detected, the feature list is emptied for further searches.

Each gait phase can be associated with a sequence of wave-related features without any complex processing that would result in a high computational load [41]. Therefore, the present algorithm conducts real-time detection while being mounted on ROS, which provides standard facilities for hardware abstraction, low-level device control, and software-architecture management [51]. The flowchart in Figure 1 summarizes this extraction process and highlights the main detection features. The feature extraction from angular velocity and linear acceleration signals starts with the creation of a feature list since various features must be found before any gait phase is claimed as detected. This list is emptied every time a new gait phase has been updated. Input data (D(i) in Figure 1: $A_{y}$ or $G_{y}$ as the case may be) are updated at a sampling rate of 100 Hz, which is in accordance with the inertial sensor sampling rate. Each feature should meet certain conditions to be recorded on the list. These conditions are sequentially evaluated as follows.

- **Crossed High Threshold (Ref. 1 in Figure 1):** Current data (D(i)) should be higher than a pre-specified threshold ($D_{high\_threshold}$) and at least 150 ms should have passed since the last saved feature. The time difference between features (Δt) is saved, along with the spotted feature into the feature list.
- **Crossed Low Threshold (Ref. 2 in Figure 1):** Current data (D(i)) should be lower than a pre-specified threshold ($D_{low\_threshold}$) and at least 150 ms should have passed since the last saved feature. Δt is also saved into the feature list.
- **Crest Middle (Ref. 3 in Figure 1):** Current data (D(i)) should be higher than a pre-specified threshold ($D_{crest\_threshold}$) and at least 150 ms should have passed since the last saved feature. Δt is also saved into the feature list.
- **Crest (Ref. 4 in Figure 1):** This feature is only assessed if *Crest Middle* has been saved into the list. Therefore, current data (D(i)) should have crossed the already exceeded pre-specified threshold ($D_{crest\_threshold}$) and a certain amount of time ($t_{crest\_threshold}$), which differs between acquired signals ($A_{y}$, $G_{y}$), should have passed since the last saved feature, so that a crest may be reported. Δt is also saved into the feature list.
- **Trough Middle (Ref. 5 in Figure 1):** Current data (D(i)) should be lower than a pre-specified threshold ($D_{trough\_threshold}$) and at least 150 ms should have passed since the last saved feature. Δt is also saved into the feature list.
- **Trough (Ref. 6 in Figure 1):** This feature is only assessed if *Trough Middle* has been saved into the list. Therefore, current data (D(i)) should again be above the already crossed pre-specified threshold ($D_{trough\_threshold}$) and a certain amount of time ($t_{trough\_threshold}$), which differs between acquired signals ($A_{y}$, $G_{y}$), should have passed since the last saved feature, so that a crest may be reported. Δt is also saved into the feature list.
- **Neutral (Ref. 7 in Figure 1):** A neutral region is only reported as long as the current data (D(i)) remains within the range between $D_{neutral\_min\_threshold}$ and $D_{neutral\_max\_threshold}$, and if a certain amount of time ($t_{neutral\_threshold}$), which differs between acquired signals ($A_{y}$, $G_{y}$), has passed since the last saved feature. Δt is also saved into the feature list.

The selection of correct threshold values was carried out as in [41], whose study validated all possible thresholds within a range, and whose limits were visually established from signals acquired in a preliminary analysis. For a trial over a flat surface, thresholds were considered optimal if the algorithm was capable of detecting a specific number of walked steps.

After each condition comprised in the feature extraction process is checked, the feature list is reviewed to determine whether a new gait phase has been encountered. A summary of the feature-based rules governing the different transitions between gait phases is given below.

**Figure 2:**
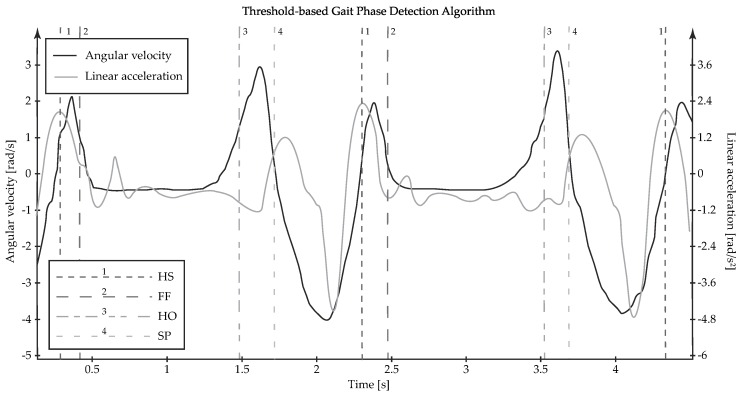
Threshold-based gait phase detection by means of an inertial sensing system over two gait cycles. Feature-based decisions are made to identify each gait phase start: HS (first dashed line), FF (second dashed line), HO (third dashed line) and SP (fourth dashed line).

- **SP → HS:** With the aim of detecting the onset of HS, the current linear acceleration data should be right in the middle of a crest, after another crest, a trough and a crossed high threshold have been sequentially entered in the feature list, whereas the angular velocity signal should have exhibited a trough and a crossed high threshold, as shown in Figure 2.
- **HS → FF:** With the aim of detecting the onset of FF, the current linear acceleration data should be right in the middle of a trough, after entering the crest corresponding to HS in the feature list, whereas the angular velocity signal should have exhibited a crest (see Figure 2).
- **FF → HO:** With the aim of detecting the onset of HO, the current linear acceleration data should remain within a neutral region for a certain amount of time, as the angular velocity signal also exhibits a neutral region, followed by a crossed high threshold (see Figure 2).
- **HO → SP:** With the aim of detecting the onset of SP, the current linear acceleration data should have crossed a pre-defined threshold, whereas the angular velocity signal should have exhibited a crest, as shown in Figure 2.

**Figure 3:**
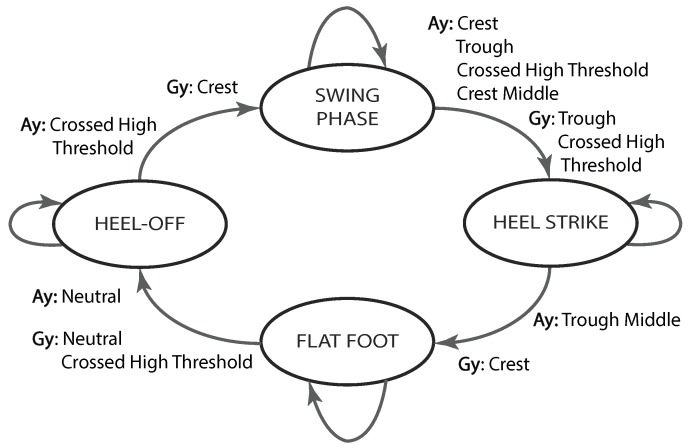
State machine of the threshold-based algorithm. The transition conditions are based on features found in the angular velocity ($G_{y}$) and linear acceleration ($A_{y}$) signals.

Figure 3 recaps the mentioned feature-based conditions for the transitions between gait phases. Similar strategies based on curve characteristics could be carried out in the case of different inertial signals drawn from different locations in the human body.

#### 3.1.2. Classification Using a Hidden Markov Model

A hidden Markov model (HMM) is a doubly stochastic process with *Q* underlying discrete states that are not observable, i.e., its state sequence is hidden to the observer who only has access to the emissions of each state [52]. The second embedded stochastic process describes such emissions, i.e., either the sensor readout or feature vectors extracted from them, in terms of discrete probabilities or probability density functions (PDFs) [44]. HMM is a statistical model widely used to estimate a sequence of hidden states in a time series [11], which for the case of gait phase detection corresponds to the gait events (Q=4).

HMM can be expressed as a function of a set λ of statistical measures:

(1) λ=(A,B,π)

which includes the probability distribution matrix of state transition *A*, the probability distribution matrix of observation symbols *B*, and the initial state distribution vector π.

**Figure 4:**
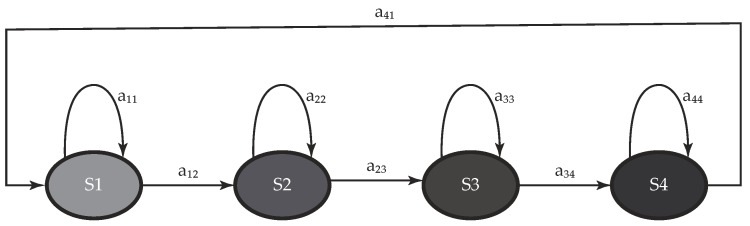
Possible transitions $\left(a_{ij}\right)$ among four states $\left(S_{i}\right)$ of a continuous HMM according to a left–right model. Each model state is paired to a gait phase, whose emissions are modeled using a Gaussian mixture model with three components. $a_{ij}$ denotes the transition probability from state $S_{i}$ to state $S_{j}$.

The normal gait pattern repeats itself indefinitely with a known sequence of gait events, which, probabilistically speaking, means that it may either remain in the current state or eventually transition to the consecutive state (see Figure 4). This behavior has been recently modeled using a left–right model [31,44], whose main feature is to limit transitions to consecutive states of the Markov chain. Since transitions represent a narrow fraction of the gait cycle, their associated probabilities assume lower values than those related to permanence in the same state. Thus, the transition matrix A may be implemented as follows [31]:

(2) $$A=\left\{a_{ij}\right\}=\left[\begin{array}{cccc} 0.9 & 0.1 & 0 & 0\\ 0 & 0.9 & 0.1 & 0\\ 0 & 0 & 0.9 & 0.1\\ 0.1 & 0 & 0 & 0.9 \end{array}\right]$$

where $a_{ij}$ denotes the transition probability from state $S_{i}$ to state $S_{j}$ (Figure 4).

Because the initial state of the model is unknown, an initial state distribution vector π that allocates the same probability to all states, i.e., each state has the same probability of being the first in a state sequence, was chosen. Finally, a bivariate GMM with three components was utilized to describe the emissions from each state. These emissions allude to feature vectors that include the angular velocity measured at any sampling time, and its time derivative computed by means of a first-order finite difference approximation, i.e., the angular acceleration [45]. This particular stochastic model yields the best trade-off between complexity and accuracy for gyroscope signals [39,44].

**Figure 5:**
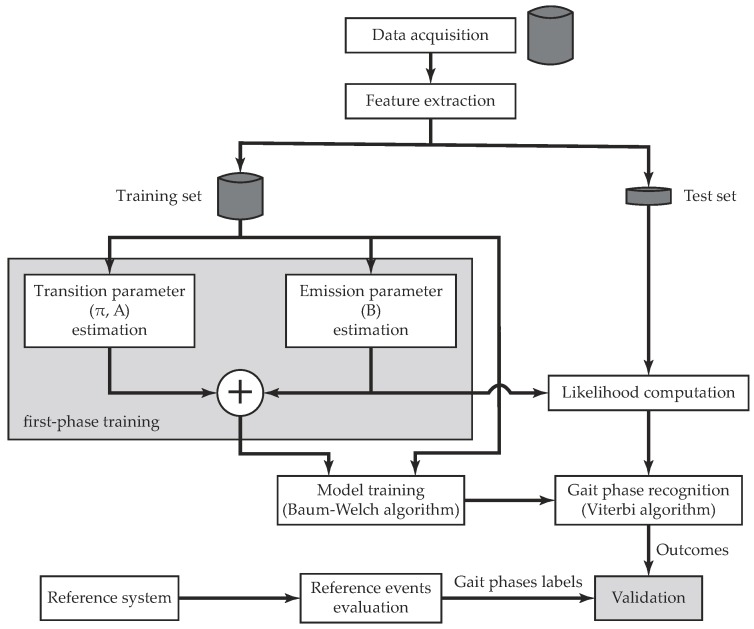
Flowchart that illustrates the validation methodology of HMM. A model is trained by means of the Baum–Welch algorithm, after applying feature extraction to the acquired dataset. The optimal state sequence is then computed through the Viterbi algorithm by using feature vectors from the test dataset, and the performance evaluation is conducted with respect to gait phases labels drawn from the reference system.

The development of a continuous HMM entails two main procedures: a training stage and a test stage. The first phase refers to the adjustment of model parameters λ to optimally adapt them to an observed training dataset [52]. The Baum–Welch algorithm, the most common solution to this issue, is implemented in the present work. This training procedure basically starts with an initial parameter set (*first-phase training* in Figure 5), on the basis of which it extracts probabilistically weighted state sequences. The initial model is repeatedly updated with these new transition and emission probabilities until a desired level of convergence is reached [39].

Subsequently, the test stage allows feature classification based on the trained model achieved in the training phase, i.e., the search for the optimal state sequence is undertaken. The most widely used optimality criterion is carried out by the Viterbi algorithm, which finds the most likely state sequence [52]. This algorithm is a dynamic programming technique that finds the optimal path by tracing back an array of likelihood indicators. The Viterbi algorithm, despite its computational efficiency, is not suitable for real-time decoding because the indicators it uses are computed starting from the end of the observed sequence of feature vectors. The validation of the classifier outputs is therefore offline compared with respect to an FSR-based reference system that provides the actual gait phases labels. The flowchart in Figure 5 summarizes the overall validation process.

For further information on HMMs, please refer to the works by Rabiner [52] and Taborri et al. [31], who provide an excellent description of what this machine-learning scheme is capable of, and whose contribution has inspired the present research.

### 3.2. Experimental Procedure

The experimental protocol was performed with the aim of quantifying the detection success ratio of the described algorithms within a varied spectrum of walking styles. To this end, eighteen participants were enrolled in this study, forming two groups: a control group with nine healthy subjects (H, 4 females, 5 males, 23.22±1.99 years old, 1.70±0.046 m, 66.87±4.29 kg), who had no known orthopedic, metabolic, or neurological impairment that could modify their natural walking pattern (see Table 1 for further information); and an experimental group with nine patients (P, 4 females, 5 males, 41.33±15.83 years old, 1.71±0.098 m, 72.28±7.73 kg), who had suffered from a hemiparesis for at least one year (see Table 2 for further information).

Despite their gait limitations, subjects from P group should be able to walk short distances without any assistive device. An additional exclusion criterion was the absence of heel contact at the IC of the gait cycle. All subjects were informed about the scope and purpose of the experiment, and written consent was obtained from each of them prior to the study. The protocol was approved by the Ethics Committee of the Colombian School of Engineering Julio Garavito, Bogota, Colombia.

**Figure 6:**
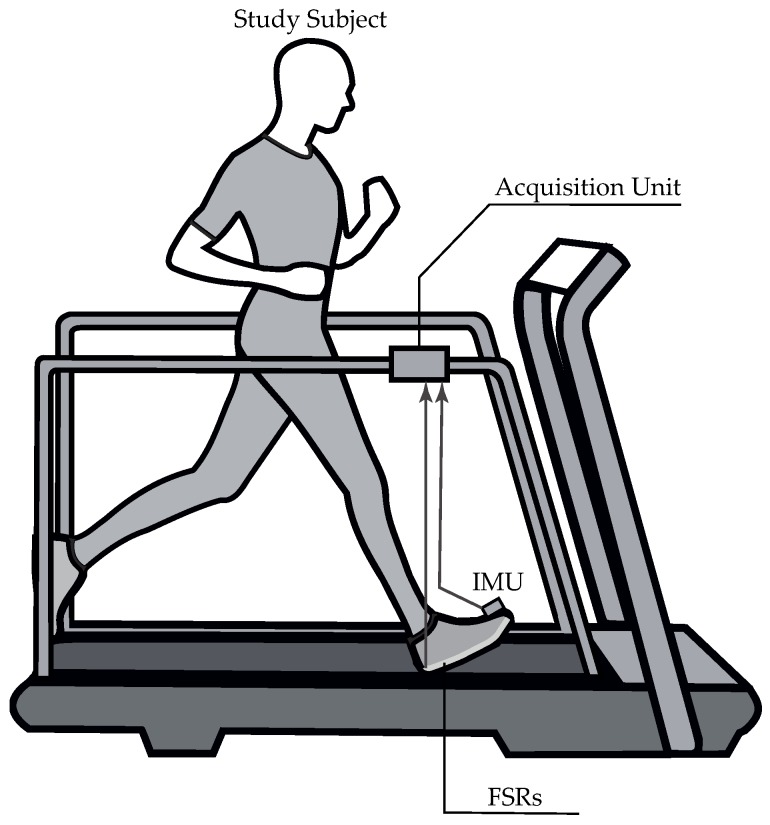
Experimental setup. Each subject was instrumented on their dominant/affected side with a custom FSR-equipped insole and an IMU placed on the dorsal side of their foot. Foot motion data were captured by using a Raspberry Pi running ROS to ensure synchronized data acquisition.

Volunteers were first instructed to perform three 10-m tests at a self-selected speed in order to determine their normal overground speed, which was successively set on a rehabilitation treadmill (NIZA RX K153D-A-3, SportFitness, Bogota, Colombia). Participants were equipped with a custom insole instrumented with FSRs (Ref. 402, Interlink Electronics, Camarillo, CA, USA) on their dominant side (for the case of H group) or their affected side (for the case of P group), which matched each subject’s shoe size and represented the reference system, and an IMU (BNO055, BOSCH, Gerlingen, Germany) placed on their foot instep. The FSR that was used consists of a conductive polymer film with a circular sensing area of 18.22 mm in diameter and a negligible thickness of 0.5 mm, while the used IMU integrates a triaxial 14-bit accelerometer, a triaxial 16-bit gyroscope, with a measurement range of $\pm 2000{\;}^{\circ }$/s, a triaxial geomagnetic sensor and a 32-bit cortex M0+ microcontroller in a single package. Special care was taken when aligning the IMU y-axis with the sagittal plane since the accelerometer signal was taken into account within the TB classification [3]. The FSR sampling rate was 200 Hz, twice as much as the one at which the IMU acquired data (100 Hz), since there must be a greater amount of reference values in order to accomplish a proper assessment of the gait phase detection algorithms. Synchronized data capture was ensured by integrating a data acquisition node in ROS, which runs on a Raspberry Pi 3 Model B that is used as the main board. The experimental setup described here is illustrated in Figure 6.

Participants were then asked to walk for at least 180 s on the already configured treadmill at a zero-degree inclination while wearing a security harness, if they considered it necessary. H group walked within a speed range of 0.639–0.944 m/s, whereas P group walked within a speed range of 0.278–0.667 m/s (Table 2). All tasks were repeated three times so that enough data to train the HMM-based method were collected. A resting period of at least 2 min was carried out between tasks to prevent fatigue. Data acquisition only started once the self-selected speed was reached, and the treadmill speed was only reduced after all data were acquired to prevent data capture during the transient state. The entire experiment, including donning/doffing times related to instrumentation procedures and walking tasks, was completed within 30 min for all volunteers. All participants completed the tasks without expressing fatigue, except for P2 presumably due to her advanced age and sedentary lifestyle.

### 3.3. Data Processing

**Figure 7:**
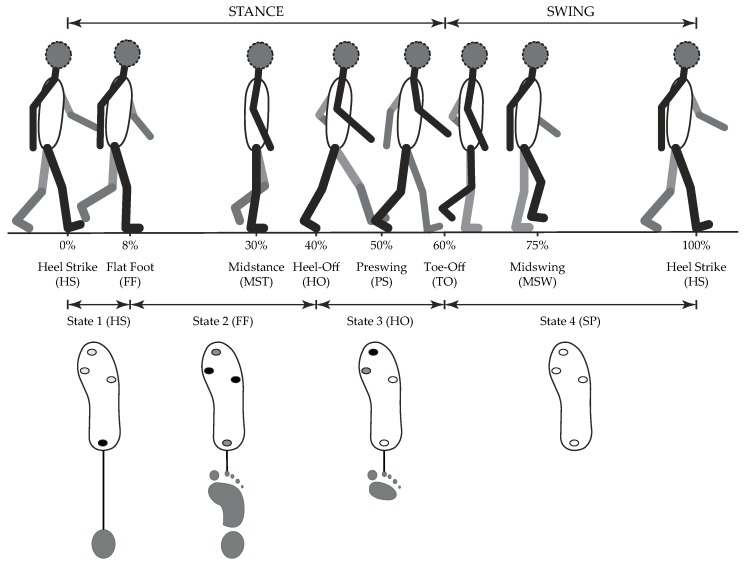
Gait phase detection based on FSR activation patterns. The gait cycle may be divided into seven phases, but four detected phases are sufficient for control purposes of an AFO. With this particular granularity, gait phases can be sequentially referenced from FSR data as follows: (i) HS State: Heel FSR is activated; (ii) FF State: first and fifth metatarsal FSR must be activated (black dots), and the activation of heel and toe FSRs is considered depending on specific study subject (grey dots); (iii) HO State: toe FSR must be activated, and the activation of first metatarsal FSR is considered depending on specific study subject; and (iv) SP State: all FSRs must be deactivated. Adapted from [21].

To assess the performance of each detection algorithm with respect to a reference system, four FSRs placed on an insole custom for each user were implemented. The exact location of these FSRs was the hallux, the first and fifth metatarsophalangeal, and the heel. The reason this distribution of pressure centers was chosen is based on how the vertical ground reaction force (GRF) shows unique pattern characteristics in these specific regions [19]. For healthy subjects, the heel experiences great pressure during the time frame between HS and midstance (MST), also known as loading response (LR), whereas the metatarsal is loaded with a great amount of pressure during the time frame between MST and Preswing (PS), also known as the terminal stance (TS), to gain forward acceleration. At the end of the gait cycle, pressure concentrates on the big toe during HO on account of the ankle plantar flexion (see Figure 7).

By means of this type of sensors, the gait phases of interest can be detected through a simple binary algorithm, since each gait phase can be related to a unique activation pattern, as can be seen in Figure 7. Along with a single stride, certain patterns of foot pressure distributions should sequentially appear. Only the heel FSR should be activated at HS since this gait phase consists of the first heel contact and the start of the gait cycle. In the case of FF, some studies suggest that all FSRs should be activated in order to detect this gait phase [21,31]. However, even though this presumption might be true for non-pathological subjects, for the treated patient sample of this work, the activation of the toe and heel FSR had to be discarded in this condition to improve the accuracy of the FSR-based gait phase detection (gray dots in Figure 7). This change was decided during offline data pre-processing from footage taken during experimental trials. Likewise, the HO occurrence depends not only on the toe FSR activation, but the first metatarsus FSR might also be taken into account depending upon offline video analysis. Finally, SP detection was given under the release of all FSRs. The FSR amplification circuit was calibrated, so that it was sensitive enough to ensure their (de)activation. The FSR accuracy was additionally assured during static trials conducted prior to the dynamic trials, in which subjects were asked to assume different standing positions to simulate various pressure patterns.

Data processing was performed offline using MATLAB software (MathWorks, 2018a, Naticks, MA, USA) and an Asus VivoBook S15 S510UA (IntelCore i5-8250U, CPU@1.80 GHz, Taipei, Taiwan) running Ubuntu 18.04. The gyroscope and accelerometer outputs were first treated with a median filter to eliminate atypical data values and a second-order low-pass filter Butterworth (cut-off frequency: 17 Hz for accelerometer signals, and 15 Hz for gyroscope signals) [31,42]. Then, inertial signals were interpolated to match the FSR frequency and partitioned into the gait phases of interest according to the previously described FSR-based segmentation logic. Finally, the partitioned angular velocity data of each gait event were time-normalized, and means and standard deviations of the obtained dataset were used to train parameters of the continuous HMM by means of the Baum–Welch algorithm.

The training stage of the HMM-based method was conducted by means of two different approaches: the well-known intra-subject procedure, hereafter referred to as subject-specific training (SST); and the inter-subject procedure, hereafter addressed as standardized parameters training (SPT). The SST model parameters were trained by means of a leave-one-out cross-validation applied to the three walking trials, whereby two trials were used for training and the remaining one for validation [31]. The cross-validation analysis was repeated in a recursive manner for each subject of both study groups so that it was repeated for all trials in turn. On the other hand, since pathological gait pattern greatly varies from one subject to another, we decided to validate an additional SPT procedure. By means of this technique, a standardized parameters set was computed on the basis of data gathered from healthy subjects, on account of the low inter-subject variability exhibited by their angular velocity waveforms [46]. A different validation approach was undertaken for each study group during SPT, in such a way that for H group, for instance, the training dataset for each examined subject was collected from the first two trials of the remaining individuals, while the last trial related to the subject under consideration was used for validation later. Conversely, for P group, the average of angular velocities acquired from healthy subjects during the first two trials was used to construct the training dataset. The final trial of each patient was subsequently involved in the validation stage [11]. Finally, for the case of the TB algorithm, all walking trials were utilized, one after the other, as the validation dataset of such a classification method.

### 3.4. Data Analysis

The performance of the proposed gait detection methods was evaluated through two indices: the timing error, i.e. the time difference of each detected gait event with respect to the FSR-based reference system, and the goodness index (*G*). *G* represents a Euclidean distance in the receiver operating characteristic (ROC) space, which poses as a global index of the classifier capability and is based on its sensibility and sensitivity values [11]. The sensitivity, also known as true positive rate (TPR), is computed as follows

(3) $$TPR=\frac{True\;Positive}{True\;Positive+False\;Negative}$$

where a true positive is considered if the classifier prediction and the reference value match within a tolerance window of 60 ms centered at each time step [32], in such a way that a gait phase was counted as detected even though it was missed by around six samples. Otherwise, such classification is considered a false positive. Likewise, the specificity, also known as true negative rate (TNR), is computed as follows

(4) $$TNR=\frac{True\;Negative}{False\;Positive+True\;Negative}$$

where the non-transitions similarly detected by classifier and reference signal correspond to true negative; otherwise, they have been accounted for by false negatives. The use of TPR and TNR metrics have some implications to bear in mind when comparing performance among classification algorithms. For instance, a gait phase detection algorithm that labels all samples of a gait trial with the same gait phase may score around 25% sensitivity and 75% specificity (with a granularity of four gait phases, assuming that each gait phase has the same time length). This sensitivity score is given because the invariant classifier output eventually matches one gait phase, whilst the specificity score comes from the fact that classifying all samples in the same way will produce true negatives for most samples [49]. Having these premises in mind, *G* is expressed as:

(5) $$G=\sqrt{{\left(1-TPR\right)}^{2}+{\left(1-TNR\right)}^{2}}$$

*G* can assume values between 0 and $\sqrt{2}$, and a classifier can be considered: (i) optimum when G≤0.25; (ii) good when 0.25<G<0.7; (iii) random if G=0.7; and (iv) bad if G>0.70 [33].

To assess the performance of the proposed classifiers in the evaluation of gait variability, mean time (MT) and coefficient of variation (CoV) of stride and each phase were determined. This calculation was undertaken for the output labels of the FSR-based reference system and each classifier. MT and std values were specifically computed for the estimated stride time and the estimated duration of each detected gait phase, for each walking task, and each study subject. CoV was then computed as:

(6) $$CoV=\frac{std}{MT}\times 100$$

### 3.5. Statistical Analysis

The software package SPSS (IBM-SPSS Inc., Armonk, NY, USA) was used for the statistical analysis. The normal distribution of all performance indices was first verified by means of the Shapiro–Wilks test. Since most data did not exhibit a normal distribution, Friedman tests were carried out to find statistically significant differences among classifiers and, where applicable, differences with respect to the reference values. Bonferroni’s tests were undertaken as a post-hoc test in case significant differences were found. An additional paired sampled t-student test was performed to find noteworthy differences between training times of the two training modalities applied to the HMM-based method. Statistical difference was a set at 0.05. Moreover, intra-class correlation coefficients (ICC) based on single-measurement, absolute-agreement, two-way mixed-effects model were computed to evaluate interrater reliability of MT and CoV values (0≤ICC≤0.4: poor reliability; 0.4<ICC<0.6: fair reliability; 0.6≤ICC<0.75: good reliability; 0.75≤ICC≤1.0: excellent reliability [31]). This reliability measure reflects the variation of data measured by one rater across more than two trials. To better interpret these ICC values, it is important to note that a low ICC could be related to the low degree of measurement agreement as well as the lack of variability among sample subjects, and the small number of subjects and the small number of raters being tested [53].

## 4. Results

Timing differences between the reference gait labels and the corresponding gait phases detected by either the TB or the HMM-based method are presented in Table 3. Almost all timing errors are negative, which indicates anticipation in the gait detection regardless of the segmentation method used. A performance improvement by the HMM-based algorithm was statistically significant for at least one training modality, i.e., either SST or SPT, for all gait phases in the case of healthy subjects (p<0.05). Gait detection for patients, on the other hand, only exhibited a significant difference between algorithms in terms of timing errors in the detection of FF.

**Figure 8:**
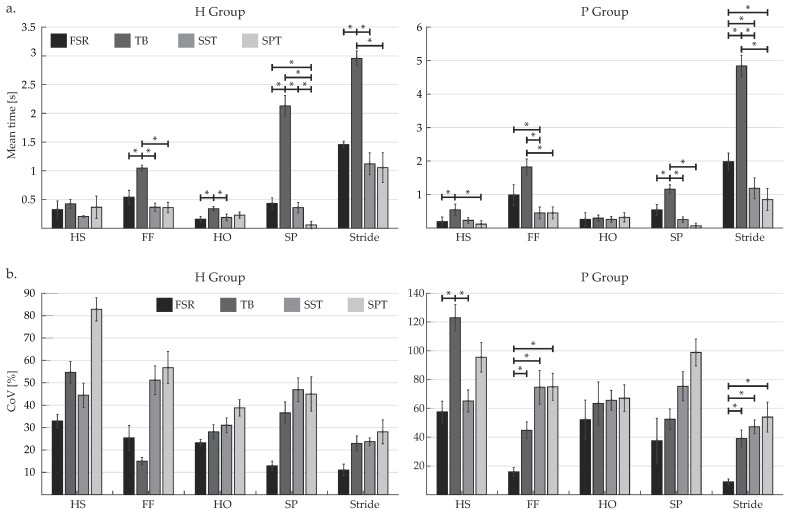
(**a**) Mean time (MT); and (**b**) Coefficient of variation (CoV) evaluated for stride and each gait phase for the reference system (FSR), and each detection algorithm: threshold-based (TB) and HMM-based algorithm, both using subject-specific training (SST) and standardized parameter training (SPT). The left bar chart corresponds to the results of healthy subjects (H), and the right graph to those of pathological subjects (P). Asterisks denote statistically significant differences (Bonferroni, p<0.05).

In Figure 8a, mean and standard deviation of MT for stride and each gait phase along with statistically significant differences (p<0.05) are reported for each detection algorithm and for the FSR reference value.

With regards to the TB algorithm, statistically significant differences were found in comparison with at least two other values (FSR- or HMM-based methods) in the evaluation of FF, HO, SP and stride MT for H group, and HS, FF, SP and stride MT for P group. Significant differences with respect to the reference labels (FSR) were additionally observed for the HMM-based algorithm trained with either a subject-specific or standardized parameters approach in the evaluation of SP MT for H group and for the evaluation of stride MT for P group. Particularly, MT assumes the lowest values for HS/HO and the highest ones for FF, which is in accordance with the event distribution associated with the normal gait cycle.

Additionally, the mean and standard deviation of CoV for stride and each detected gait phase together with statistically significant differences (p<0.05) are shown in Figure 8b for FSR and each detection algorithm. With regard to the group of healthy subjects (H), no statistically significant differences were found among FSR and classifiers, whereas differences between FSR and at least one classifier were noticeable in the evaluation of HS, FF and stride CoV for pathological subjects (P).

Values of ICC for MT and CoV are included in Table 4. The results indicate that ICC was almost always in the range of good or excellent reliability, with the exception of a few values related to the TB algorithm that showed poor reliability.

Table 5 shows the confusion matrices obtained for each proposed classification method (TB, SST and SPT) considering both study groups (H and P). For the case of the HMM-based algorithms, misclassification mostly occurred during consecutive gait phases, i.e., during phase transitions. For instance, the FF identification by using the SST method in healthy subjects exhibits 5.02% and 6.42% of samples being misclassified as HS and HO, respectively, whereas only 2.75% of FF samples were classified as SP. The confusions between two successive phases can be explained by the fact that the reference labels, which are defined manually, may be subject to labeling errors, particularly in phase transitions. Conversely, the confusion matrix corresponding to the TB algorithm highlights an unusual behavior regarding FF and SP identification since the correct classification of the one presents a significant bias toward the other for both study groups.

**Figure 9:**
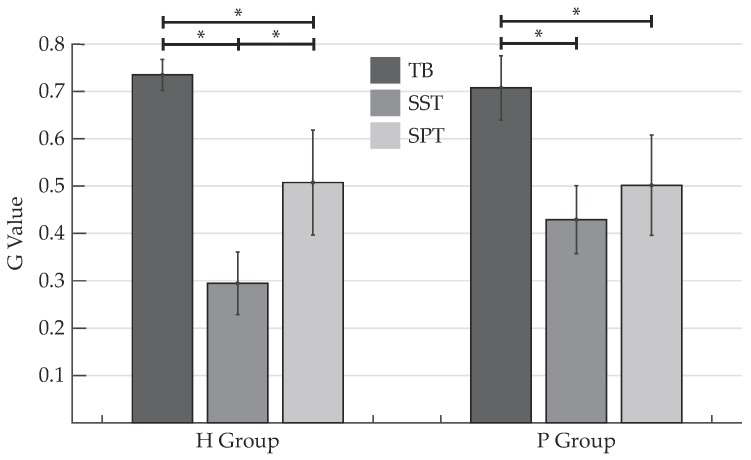
Goodness index (G) for healthy subjects (H) and subjects with mobility impairments (P) for each detection algorithm: threshold-based (TB), and HMM-based algorithm, using subject-specific training (SST) and standardized parameter training (SPT). Asterisks indicate statistically significant differences among classifiers (Bonferroni, p<0.05).

In Figure 9 the mean values and standard deviations of the Goodness index (G) for both H and P groups are reported. In addition to that, statistically significant differences among classifiers are included. The TB algorithm presented not only mean values of G that were found to be in the bad range (G>0.7) for both H and P group, but also significant differences compared to both training modalities of the HMM-based detection method. As for this latter detection algorithm, their G values were found to be in the good range (0.25≤G≤0.7) for both study groups, with the best classification performance presented by the classifier applied to data gathered from H group and trained using intra-subject cross-validation (SST). Comparing the SST and SPT procedures, significant differences between the two training approaches were only observed for the H group. The standard deviations were found to always be less than 0.2, with higher values reached by the HMM-based algorithm trained by means of standardized parameters.

By comparing both training procedures of the HMM-based detection algorithm in terms of training time, a significant difference between the SST (165.32±46 s) and the SPT approach (75.95±24 s) when applied to the P group was found.

## 5. Discussion

In this work, the validation of two algorithms: a threshold-based (TB) and a HMM-based method, in the gait event detection of healthy subjects (H group) and patients with gait abnormalities (P group) is addressed, with the objective of figuring out which detection method outperforms for a GPDS that only makes use of an IMU placed on the subject’s foot instep. The TB algorithm takes the foot angular velocity and linear acceleration along the sagittal plane as inputs, since the data fusion of these parameters allows the compensation of the drift error [12] and the use of a single-axis angular velocity signal may cause problems in situations where the subject spontaneously changes direction or turns [3]. When implementing only the HMM-based technique, it is not mandatory to take any special care to align the IMU sensitive axis perpendicular to the sagittal plane or the use of a Kalman filter for the effects induced by magnetic distortions [44], because only gyroscope signals are fed to this machine-learning scheme. The training stage used in this study is undertaken by implementing two different approaches: an intra-subject or subject-specific training (SST) and an inter-subject approach that trains the gait detection model based on standardized parameters from the dataset of the healthy group (SPT).

The TB detection method exhibited similar time differences, compared to reference values established through the FSR insole-mounted system, to those found by Rueterbories et al. [3], whose study involved both healthy and hemiparetic participants wearing biaxial accelerometers on their foot. Furthermore, compared with the findings of Mannini and Sabatini [44], our TB algorithm presents fewer time differences in the detection of stance subphases, i.e. FF and HO, for healthy subjects. However, other studies, which utilized only one inertial sensor, e.g. an uniaxial gyroscope on either the user’s shank or foot instep [23,40] or an uniaxial accelerometer on the lower trunk [43], showed smaller time delays in a two-phase model (Table 3), thereby implying a better performance than the one achieved by the TB algorithm described here. It is important to keep in mind that these results were yielded even though this method was developed based on feature extraction procedures used in [3,40], but applied to foot angular velocity and linear acceleration signals. In addition, it should be noted that the gait phase detection by footswitches such as FSRs depends significantly on the choice of threshold level that is applied to their output signals, thus producing small differences when applying different threshold settings [42]. Compared to the machine-learning method, HMM outperformed the TB method by showing significant differences with smaller timing errors for all gait phases in healthy subjects either using SST or SPT, as found by Mannini and Sabatini [44], Mannini et al. [46], whereby they also compared TB and HMM-based algorithms. For the case of hemiparetic subjects, only the detection of FF showed a significant difference among classifiers, which is an important gait event used to avoid the occurrence of false positives in the control system of a robotic orthosis while its user remains in an idle state [31].

From the analysis of MT values related to HS, FF, HO and SP, it is seen that the percentage of time spent by each gait event during the stride is in accordance with the results reported by Taborri et al. [31]. In particular, HS has the smallest duration for most cases, whereas FF exhibits the largest time length. In the evaluation of MT values, the TB algorithm yielded statistically significant differences with respect to both FSR reference values and HMM-based method since it presented numerous atypical values in the detection of different gait events for both healthy and hemiparetic subjects (see Figure 8a). Conversely, no significant differences were found between FSR-related values and the HMM-based classifier apart from the detection of SP in healthy subjects and stride in pathological ones when the model was trained by means of SPT. These differences might be explained by the fact that the data from healthy subjects with which the model parameters were improved have a wider and higher range of gait speeds compared to those subjects with mobility impairments walked (as shown in Table 1). Moreover, the analysis of ICC validates the excellent reliability of the HMM-based method trained through SST in the evaluation of gait variability based on MT (Table 4). ICC values also confirm the precarious reliability of the HS detection by using the TB threshold and the SP detection by means of the HMM-based method trained through SPT, since they exhibited results in the poor range.

Regarding CoV values, no significant differences were found between classifiers and FSR for H group and CoV values for all gait phases decreased as the gait event duration increased (as found by Liu et al. [54]). Despite this, Stride CoV for both study groups was characterized by unusually higher values in comparison to those found in the literature for healthy adults whose CoV values were not greater than 4% for walking tasks [55]. This outcome, along with lower ICC values and significant differences found between FSR and at least one classifier in the evaluation of HS, FF and stride CoV for P group, confirms the lower reliability of CoV-based assessment compared to other stride duration variability parameters [31]. These findings also imply that neither this particular TB algorithm nor a scalar HMM could be involved in studies of gait variability that require the analysis of CoV.

The classification based on the TB algorithm was only partially successful in solving the classification problem regarding the sample-based accuracy values (overall accuracy equivalent to 63.96% and 65.43% for H and P group, respectively). The significant bias presented in the identification of FF and SP (see Table 5) appears to be the reason for the poorer performance of the TB algorithm that we implemented compared to those of previous studies [3]. Such misclassifications could be related to the fact that the feature corresponding to the gyroscope data is the same for the previously mentioned phase transitions, or to the fact that the time limit corresponding to the accelerometer-related features might be too high to accurately detect FF in higher walking speeds (150 ms in contrast to the typical IC duration for healthy subjects, which is approximately 120 ms [56]). A plausible solution for this issue might be to include further features related to the angular velocity signal to improve its accuracy and to better establish the initial temporal thresholds related to accelerometer signals. As for the HMM-based partitioning techniques, in healthy subjects, the SST approach seems to outperform the SPT procedure with a higher overall accuracy rate (81.44% against 76.91%), while, for P group, the accuracy values are quite similar (78.06% for the former, and 76.36% for the latter). On the whole, FF and SP gait phases had better accuracy rates for most segmentation methods, which may be underpinned by the percentage they encompass within the gait cycle, and, therefore, the greater amount of data that is fed to the GPDS. On the other hand, the SPT approach presents a noteworthy decrease in terms of accuracy rate for the SP detection with respect to the SST procedure, which indicates that the dataset of standardized parameters drawn from H group might not be representative enough to identify this transition with high accuracy.

Finally, in regards to the goodness index (G), which involves both specificity and sensibility values in order to precisely evaluate the classifier performance, it is worth noting that the TB algorithm showed results within the bad range for both healthy subjects and participants with impairments, which, taking into account the already analyzed results, makes it the worst classifier for gait phase detection. For the case of HMM-based methods, on the other hand, G values within the good and excellent range were observed for both study groups, in spite of some high values of standard deviation exhibited in the SST timing error results (see Table 3). Even though a significant difference was found between training techniques for H group, which validates the presumption formulated on the basis of the accuracy values, the inter-subject approach based on standardized data drawn from healthy subjects still has an acceptable segmentation performance (see Figure 9). On the contrary, the training procedures did not exhibit any noteworthy difference when applied to data drawn from patients, which implies that similar and even better results may be achieved by an inter-subject trained model, as was reported in [11,29,33]. Apart from that, SPT outperforms SST in terms of training time, since there was a significant difference in the amount of time spent in the training stage between HMM-based classifiers. Therefore, the use of standardized parameters extracted from healthy subjects seems to lead to a sufficiently robust trained model that may even match the performance achieved by models constructed with SST, without the necessity of continuously carrying out a training stage. Better outcomes than the ones presented here, especially for the case of SP detection, might be obtained if the age and anthropometric parameters of pathological subjects were matched with those of healthy subjects (as was done in the previously mentioned studies), and overground walking tasks were performed, since there are some constraints inherent to treadmill walking that may modify the lower-limb kinematics [57].

There are some additional limitations to this study. For instance, since the TB algorithm takes accelerometer signals as an input, this method is dependent on the placement of the sensitive axis of the IMU, which leads to the need of having an expert operator precisely positioning the sensors on the subject. Another disadvantage of the rule-based technique is its tendency to overperform, i.e., the detection of premature transitions (see Table 3). A reason for this behavior might be irregular gait cycle times [3], and a plausible solution could be the implementation of self-tuning procedures such as the one proposed in [8]. In doing so, the difficult process of threshold tuning could also be avoided, which represents the main limitation of TB methods [17] since it is carried out empirically in most cases (including the present one). Further, the reference detection system in this study relied on footswitches. Even though foot-mounted sensors have numerous advantages such as low cost and straightforward signal processing, their accuracy may be affected by interference associated with wired connections and erroneous placement [22]. Despite these drawbacks, for applications that involve gait phase detection, the system proposed here, consisting of a single-axis gyroscope on the foot instep, not only has practical advantages in terms of aesthetics, research expenditure, ease of placement and donning/doffing times, but also poses an accurate GPDS for the control system of a robotic AFO by using an HMM-based algorithm, which avoids the time-consuming process of threshold setting.

## 6. Conclusions

This paper features the validation of two gait phase detection algorithms in a system which only involves the inertial sensing from a foot-mounted IMU: a TB and an HMM-based algorithm; the latter is trained by means of two modalities, namely an intra-subject approach and an inter-subject procedure based on standardized data from healthy subjects. Results in terms of timing errors and goodness index indicate that the best classifier is the HMM-based method, whose standardized parameter training showed similar performance rates to those exhibited by the intra-subject technique in pathological subjects. Moreover, considering the observed performance with respect to FSR, the scalar HMM trained by means of an intra-subject approach and based on MT values also represents a useful tool for assessing gait variability in both healthy and pathological subjects. Such findings suggest that the use of a single gyroscope on the dorsal side of the foot is sufficient to accurately detect gait phases within the control system of a lower-limb orthotic device. For future works, the real-time implementation of the HMM-based detection scheme on a robotic AFO is intended, and for this, the adoption of an online hidden-state decoding method such as the short-time Viterbi algorithm [46] is expected to be deployed. In doing so, we expect to be able to compare both algorithms in terms of latency, i.e., the time needed to online detect each gait phase, since the practical implications of the timing errors in biofeedback such as assistive devices have not yet been addressed. Additionally, given the fact that the proposed detection algorithms were only tested on healthy and hemiparetic individuals, further research may be aimed at the dataset extension by including subjects affected by other neurological diseases, e.g., SCI, Parkinson disease, or multiple sclerosis.

## Figures and Tables

**Table 1:** Summary of healthy participants (H group). BMI, body mass index.

Subject	Age [Years Old]	BMI [kg/m${}^{2}$]	Gender	Walking Speed [m/s]
H1	23	22.2	Male	0.944
H2	22	20.9	Female	0.750
H3	22	22.7	Male	0.750
H4	23	24.1	Female	0.694
H5	22	23.7	Male	0.694
H6	20	26.0	Female	0.639
H7	25	20.7	Female	0.556
H8	25	23.7	Male	0.833
H9	27	23.7	Male	0.833

**Table 2:** Summary of hemiparetic individuals who participated in the study (P group). BMI, body mass index.

Subject	Age [Years Old]	BMI [kg/m${}^{2}$]	Gender	Etiology	Paretic Side	Year of Ocurrence	Walking Speed [m/s]	Walking Aids
P1	37	24.7	Female	Ischemic Stroke	Left	2016	0.417	Cane
P2	75	20.1	Female	Ischemic Stroke	Right	2016	0.306	Cane or Wheelchair
P3	49	26.9	Female	Ischemic Stroke	Left	2010	0.278	AFO
P4	38	25.0	Male	Hemorrhagic Stroke	Left	2017	0.639	None
P5	47	34.0	Male	Ischemic Stroke	Left	2016	0.444	AFO
P6	33	22.0	Female	Hemorrhagic Stroke	Right	2012	0.417	Cane
P7	46	31.2	Male	Ischemic Stroke	Right	2001	0.417	Cane
P8	20	21.7	Male	Cerebral Palsy	Left	1998 (Birth)	0.5	None
P9	35	23.4	Male	Hemorragic Stroke	Left	2013	0.667	None

**Table 3:** Timing errors of the detected gait phases (mean ± std) [ms]. These data were generated by comparing the estimates provided by each classifier with the reference data. Asterisks * and obelisks ${}^{†}$ indicate significant differences between marked algorithms (Bonferroni, p<0.05).

	H Group			P Group		
	TB Method	SST Method	SPT Method	TB Method	SST Method	SPT Method
HS	-37±101 *	-55±98 *	-17±10	-17±20	-22±16	-20±17
FF	-57±102 *${}^{†}$	-57±97 *	$-23\pm 3{\;}^{†}$	-28±12 *${}^{†}$	-29±12 *	$-29\pm 12{\;}^{†}$
HO	13±30 *	-36±62	-18±6 *	9±29	-19±18	-19±19
SP	-49±96 *	-58±108 *	-18±13	-24±15	-27±14	-23±19

**Table 4:** Values of Intra-class Correlation Coefficients (ICC) for Mean Time (MT) and Coefficient of Variation (CoV) evaluated for stride and each detected gait phase by FSR reference system and classifiers.

			ICC				
Group	Index	Reference/Classifier	HS	FF	HO	SP	Stride
H	MT	FSR	0.987	0.99	0.98	0.99	0.92
		TB	0.37	0.82	0.88	0.98	0.98
		SST	0.81	0.84	0.83	0.84	0.88
		SPT	0.66	0.88	0.62	0.04	0.77
	CoV	FSR	0.95	0.99	0.60	0.95	0.94
		TB	0.69	0.75	0.62	0.90	0.18
		SST	0.87	0.90	0.92	0.80	0.82
		SPT	0.80	0.89	0.74	0.34	0.95
P	MT	FSR	0.97	0.99	0.99	0.98	0.99
		TB	0.29	0.94	0.79	0.98	0.99
		SST	0.75	0.82	0.76	0.85	0.84
		SPT	0.81	0.91	0.79	0.16	0.84
	CoV	FSR	0.86	0.70	0.97	0.97	0.75
		TB	0.94	0.83	0.84	0.87	0.93
		SST	0.75	0.79	0.67	0.95	0.80
		SPT	0.92	0.73	0.53	0.38	0.72

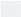
 Poor 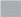 Fair 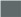 Good 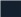 Excellent.

**Table 5:** Confusion matrices. Results are reported for each proposed classification method: (1) TB algorithm; (2) HMM-based method trained by means of an SST technique; and (3) HMM-based method trained by means of an SPT technique, for both healthy subjects (H group) and patients (P group). Correct classifications are in bold and overall accuracy values with 95% confidence interval (CI) are presented for each partitioning algorithm in each study group.

			Classification Output			
			HS	FF	HO	SP
*1. TB algorithm*						
**Actual Label**	**H**	**HS**	**57.11%**	16.59%	5.58%	20.72%
		**FF**	1.03%	**72.08%**	0.47%	26.42%
		**HO**	1.50%	18.77%	**52.98%**	26.75%
		**SP**	13.72%	7.5%	12.62%	**66.16%**
	Overall accuracy: **63.96%**. 95% CI: **63.91**–**64.01%**					
	**P**	**HS**	**57.82%**	18.24%	4.17%	19.77%
		**FF**	2.91%	**70.24%**	0.77%	26.08%
		**HO**	3.48%	20.1%	**53.13%**	23.29%
		**SP**	10.71%	13.77%	8.19%	**67.33%**
	Overall accuracy: **65.43%**. 95% CI: **65.38**–**65.48%**					
*2. HMM-based algorithm with SST approach*						
**Actual Label**	**H**	**HS**	**70.15%**	12.08%	7.22%	10.55%
		**FF**	5.02%	**85.81%**	6.42%	2.75%
		**HO**	4.40%	5.85%	**81.57%**	8.18%
		**SP**	5.33%	2.05%	2.61%	**90.01%**
	Overall accuracy: **81.44%**. 95% CI: **81.40**–**81.48%**					
	**P**	**HS**	**67.22%**	10.65%	9.2%	12.93%
		**FF**	6.44%	**81.87%**	9.39%	2.30%
		**HO**	5.86%	9.36%	**77.28%**	7.50%
		**SP**	11.66%	2.31%	7.20%	**78.83%**
	Overall accuracy: **78.06%**. 95% CI: **78.02**–**78.10%**					
*3. HMM-based algorithm with SPT approach*						
**Actual Label**	**H**	**HS**	**75.55%**	9.79%	2.92%	11.74%
		**FF**	7.29%	**85.85%**	5.98%	0.88%
		**HO**	2.19%	4.05%	**90.55%**	3.21%
		**SP**	24.76%	0.58%	13.19%	**61.47%**
	Overall accuracy: **76.91%**. 95% CI: **76.84**–**76.99%**					
	**P**	**HS**	**63.50%**	14.89%	4.78%	16.92%
		**FF**	2.66%	**84.97%**	11.33%	1.04%
		**HO**	1.49%	12.37%	**81.69%**	4.45%
		**SP**	16.33%	2.96%	17.45%	**63.26%**
	Overall accuracy: **76.36%**. 95% CI: **76.29**–**76.43%**					

## Abbreviations

The following abbreviations are used in this manuscript:

FES	Functional Electrical Stimulation
IC	Initial Contact
HS	Heel Strike
FF	Flat Foot
HO	Heel-Off
SP	Swing Phase
TO	Toe-Off
AFO	Ankle-Foot Orthosis
ENG	Electroneugram
EMG	Electromyography
FSR	Force-Sensitive Resistor
IMU	Inertial Measurement Unit
GPDS	Gait Phase Detection System
TB	Threshold-Based
HMM	Hidden Markov Model
SVM	Support Vector Machine
LDA	Linear Discriminant Analysis
GMM	Gaussian Mixture Model
ANN	Artificial Neural Network
MS	Mid-Swing
SPT	Standardized Parameters Training
ROS	Robot Operative System
FSM	Finite State Machine
PDF	Probability Density Function
BMI	Body Mass Index
GRF	Ground Reaction Force
LR	Loading Response
MST	Midstance
PS	Preswing
SST	Subject Specific Training
SPT	Standardized Parameters Training
CI	Confidence Interval
G	Goodness Index
ROC	Receiver Operating Characteristic
TPR	True Positive Rate
TNR	True Negative Rate
MT	Mean Time
CoV	Coefficient of Variation
std	Standard Deviation
ICC	Intra-class Correlation Coefficient
SCI	Spinal Cord Injury
